# Cardiac Cell Senescence and Redox Signaling

**DOI:** 10.3389/fcvm.2017.00038

**Published:** 2017-05-29

**Authors:** Daniela Cesselli, Aneta Aleksova, Sandro Sponga, Celeste Cervellin, Carla Di Loreto, Gianluca Tell, Antonio Paolo Beltrami

**Affiliations:** ^1^Department of Medicine, University of Udine, Udine, Italy; ^2^Cardiovascular Department, Azienda Sanitaria Universitaria Integrata di Trieste, University of Trieste, Trieste, Italy; ^3^Cardiothoracic Surgery, Azienda Sanitaria Universitaria Integrata di Udine, Udine, Italy

**Keywords:** aging, heart failure, reactive oxygen species, redox signaling, stem cells, APE/Ref-1, metabolism

## Abstract

Aging is characterized by a progressive loss of the ability of the organism to cope with stressors and to repair tissue damage. As a result, chronic diseases, including cardiovascular disease, increase their prevalence with aging, underlining the existence of common mechanisms that lead to frailty and age-related diseases. In this frame, the progressive decline of the homeostatic and reparative function of primitive cells has been hypothesized to play a major role in the evolution of cardiac pathology to heart failure. Although initially it was believed that reactive oxygen species (ROS) were produced in an unregulated manner as a byproduct of cellular metabolism, causing macromolecular damage and aging, accumulating evidence indicate the major role played by redox signaling in physiology. Aim of this review is to critically revise evidence linking ROS to cell senescence and aging and to provide evidence of the primary role played by redox signaling, with a particular emphasis on the multifunctional protein APE1/Ref in stem cell biology. Finally, we will discuss evidence supporting the role of redox signaling in cardiovascular cells.

## Introduction

In humans, chronological aging is associated with both a progressive decline of the ability of the organism to maintain homeostasis and with a gradual reduction of the ability to cope with stressors and to repair tissue damage. This progressive functional decline eventually leads to a condition named frailty that is characterized by an increased vulnerability to serious adverse clinical outcome triggered by minor stressor events (1). Frailty is nowadays considered to be the result of loss of homeostatic control in several physiological systems (e.g., endocrine, hematopoietic, skeletal muscle, nervous, and cardiovascular systems) and has been associated with altered energetics (2). This condition may occur even in the absence of specific pathologic conditions. However, chronic diseases, such as heart failure, type II diabetes, and chronic obstructive lung disease, increase their prevalence with aging and increase the risk for frailty [reviewed by Ref. (3)]. To understand the common mechanisms that lead to frailty and age-related diseases is a challenge that may unveil strategies for rejuvenation and that could increase the health-span. According to current theories, aging would result from the interaction between environmental stresses and error-preventing or error-correcting systems that are comprised within a finite organism, where the majority of energy is allocated to maintain the reproductive system at the expenses of the soma [discussed in Ref. (4)].

Among age-related diseases, cardiovascular disease has an impressive prevalence, considering that the remaining lifetime risk for cardiovascular disease is about 50% at the age of 40 (5). Consistently, the pathophysiologic modifications that are observed in aging hearts and arteries interact with alterations that characterize atherosclerosis progression, concurring to the development of age-associated heart failure. This latter is due to a combined diastolic and systolic dysfunction, caused by cardiac hypertrophy, replacement fibrosis, and myocardial ischemia, even in the absence of atherosclerotic coronary disease (6).

## (Stem) Cell Senescence in Cardiovascular Aging and Heart Failure

Morphometric data acquired in the early 1990s suggested that the number of left ventricle cardiomyocytes declines progressively with aging (7). Consistently, investigators have documented that although cardiomyocyte turnover occurs postnatally, the rate of cardiomyocyte renewal declines as age advances (8). Intriguingly, while the same investigators have recently suggested that the total number of cardiomyocytes residing in the left ventricle does not change with aging (9), evidence of myocyte death has been shown to occur both in male primates (10) and in humans. In these latter, cardiac troponin T levels increase with aging and can predict cardiovascular events and death in the general population (11, 12). This finding is thought to be the consequence of the age-related reduction of expression or activity of proteins that are involved in cardioprotection, a condition that eventually leads to an increased susceptibility of cardiac myocytes to injury [reviewed in Ref. (13)].

To understand the mechanisms leading to heart failure, we and other authors hypothesized that the reduced cardiomyocyte turnover observed in aging was a consequence of the reduced cardiac growth reserve. Although, as anticipated, postnatal cardiomyocyte proliferation has been widely accepted, a heated debate surrounds the origin of these mature cells. As we discussed in a recent review (14), several independent groups have shown that undifferentiated, primitive cells reside in mammalian hearts and are involved in cardioprotection against heart failure, possibly generating new myocytes (15). Conversely, different lines of evidence obtained in animal models of heart failure (e.g., doxorubicin-induced cardiomyopathy, diabetic cardiomyopathy) and in humans (i.e., patients affected by heart failure of ischemic and non-ischemic nature) indicate that senescent and dysfunctional cardiac resident stem/progenitor cells (CS/PC) accumulate as a consequence of cardiac pathology (16–20). Furthermore, with organism aging, senescent primitive and differentiated cells accumulate in mammalian hearts (21, 22).

Although the concept of cellular senescence was introduced more than 50 years ago, postulating that the irreversible growth arrest observed in cultures of diploid human cells after a fixed number of cell replications was one of the main mechanisms of organism aging, the debate around this programmed cellular behavior is still ongoing (23). Specifically, in relatively recent years, it has been shown that cell senescence may exert positive effects, by promoting tissue healing after injury and protecting young organisms from cancer (24). However, in line with the antagonistic pleiotropy theory of aging, these beneficial effects exerted by cell senescence in young animals may be also responsible for the occurrence of functional impairment and age-related pathologies (25). Consistently, “rejuvenation” strategies aimed at reducing the frequency of senescent cells in the organism or designed to modulate those pathways whose activation status is altered in cell senescence can restore cardiac function in aged and failing hearts (19, 22).

Finally, we should emphasize that, while it has been postulated that reactive oxygen species (ROS) play a primary role in the development of cell senescence (26, 27), the molecular mechanisms responsible for the development and evolution of cellular senescence are still a matter of intense research.

## Involvement of ROS in Aging and Cell Senescence

In this regard, for many years it was believed that ROS were produced in an unregulated manner as a byproduct of cellular metabolism. Moreover, their ability to cause damage to macromolecules (e.g., proteins and nucleic acids) was thought to be responsible for organism aging (also known as the mitochondrial free radical theory of aging -MFRTA-) (28). Consistently, several pieces of evidence have shown an age-dependent decrease in mitochondrial integrity, and a parallel increase in the level of oxidized DNA (including the mitochondrial one) (29, 30). These alterations have led to the formulation of “the vicious cycle hypothesis of mitochondrial ROS generation,” according to which the mitochondrial production of ROS would damage mitochondrial DNA (mtDNA) and lead to mitochondrial dysfunction, thus increasing ROS generation. However, discordant results have been obtained in more recent years, which have either supported or refuted the increased production of mitochondrial ROS with aging (31–33). Most importantly, the experimental modulation of intracellular ROS levels led to contradictory effects on animal lifespan. Among the studies that supported the free radical theory of aging, we should acknowledge that: the deletion of p66^shc^, an isoform of the proteins encoded by the SHC1 gene that modulates mitochondrial ROS generation in response to stress activated p53 (34), determined the extension of lifespan in mice (35). Additionally, by targeting human catalase, an enzyme that is normally localized within the peroxisome, to either the nucleus or the mitochondria of transgenic mice, authors described a significant increase of median life span only in the latter group of animals (36). However, a series of experimental studies have provided strong evidence against the core of the MFRTA. Among these, the unsuccessful or even deleterious effects of the exogenous supplementation of antioxidants that was observed in clinical trials (37) or the genetic manipulation of the levels of manganese-dependent superoxide dismutase (SOD2, the major mitochondrial superoxide scavenger) that failed to show effects on mouse life span (38, 39).

These seemingly contradictory results can be reconciled if we consider that ROS have a dual nature, as recently reviewed in Ref. (40). In fact, on top of their ability to damage in non-specific fashion biological molecules, ROS (specifically ${\text{O}}_{2}^{\cdot -}$ and H_2_O_2_) can exert useful and beneficial effects, by regulating signaling pathways. Although the modalities of reduction-oxidation (redox)-dependent signaling have been only partially delineated (see discussion later), according to current models, ROS generation is highly regulated and redox-dependent transduction pathways are compartmentalized. Therefore, oxidative stress would arise from the loss of this architecture (41). Given these premises, we will discuss how the sources of oxidants and the mechanism of redox signaling are related to cell senescence.

### Oxidant Sources that May Be Altered in Cell Senescence

Although in most cell types mitochondria are considered to be the major producers of ROS, other modalities of oxidant generation are also in place. We will briefly discuss them, pointing out their involvement in cellular senescence processes.

**Figure 1:**
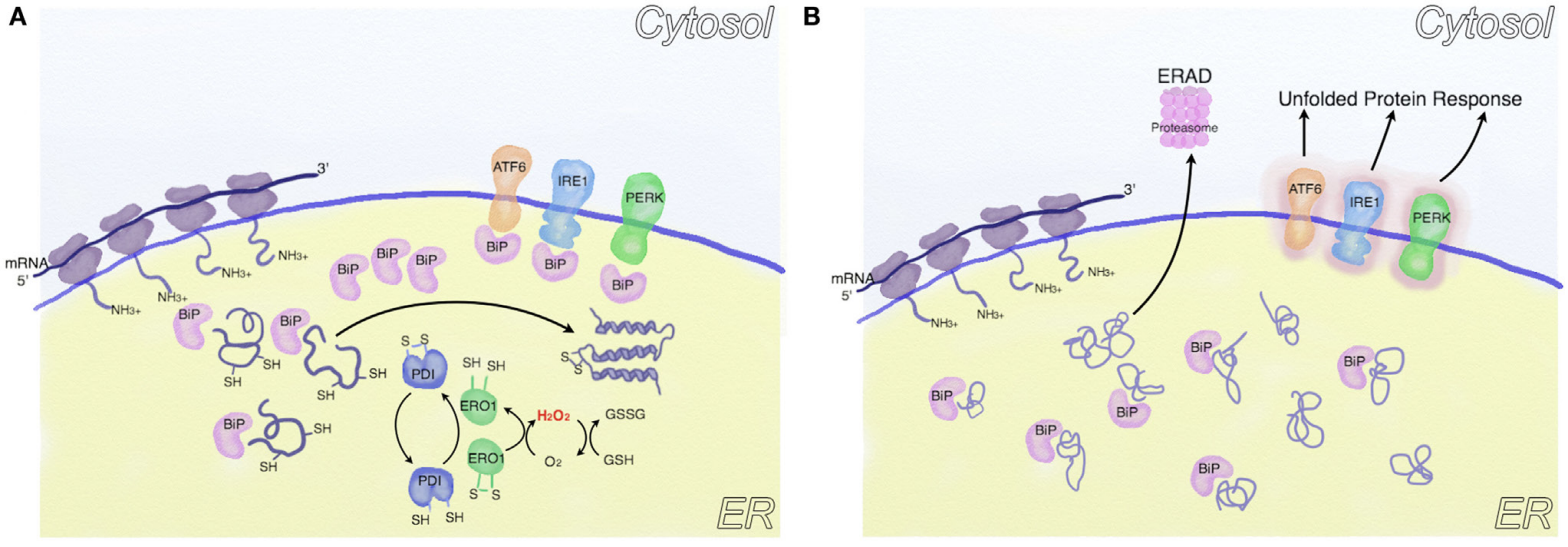
**Oxidative folding and endoplasmic reticulum (ER) stress**. **(A)** Following protein synthesis, the activity of chaperones (such as BiP) and ER enzymes is coordinated to reach a correct protein folding. In this process, protein disulfide isomerase (PDI) oxidizes critical cysteine residues in nascent proteins to facilitate correct folding, resulting in PDI reduction. PDIs are subsequently oxidized [mainly by endoplasmic reticulum oxidoreductin 1 (ERO1)], thus generating H_2_O_2_, which is buffered by cytoplasmic glutathione. **(B)** In conditions associated with ER stress (e.g., increased protein synthesis or altered ER redox balance), unfolded proteins accumulate in the ER lumen. Part of these are sent to ER-associated degradation, part sequester BiP, thus activating the unfolded protein response (UPR).

The *endoplasmic reticulum (ER)* is an organelle responsible for lipid, steroid and protein biosynthetic processes, protein post-translational modifications (e.g., glycosylation and disulfide bond formation), protein folding, and Ca^2+^ storage (42). ER-localized enzymes and chaperones (most prominently BiP) work together to facilitate protein folding. Intriguingly, oxidative folding (i.e., the process of correct protein folding and disulfide bond formation, Figure 1), which requires the transfer of one electron from protein disulfide isomerase (PDI) to endoplasmic reticulum oxidoreductin-1 and molecular oxygen, provides a link between protein synthesis and ROS generation (43). Furthermore, a correct balance between reduced and oxidized glutathione is required to maintain ER oxidoreductases in a reduced state and to buffer the ROS produced by the oxidative folding process (44). However, when proteins fail to fold, they are sent to ER-associated degradation (ERAD) via the ubiquitin proteasome system (45). Importantly, certain conditions, including an altered balance of reducing and oxidative agents (46, 47) or an increased rate of protein synthesis that saturates the folding capacity of the ER, eventually lead to the accumulation of unfolded proteins in the ER lumen, causing ER stress (45). Prolonged ER stress both activates the unfolded protein response (UPR), which either restores cell homeostasis or induces apoptosis, and promotes ROS generation (45). In line, UPR and ROS can activate apoptosis signal regulating kinase 1 (ASK1), an oxidative stress responsive kinase, that leads to the activation of p38 mitogen-activated protein kinase (MAPK) and c-Jun-N terminal kinase (JNK) (48). Another possible source of ER-derived ROS are proteins of the reduced nicotinamide adenine dinucleotide phosphate (NADPH) oxidase (NOX) family, whose members form protein complexes that catalyze the transfer of electrons from NADPH to molecular oxygen to produce superoxide anions in different physiologic and pathologic conditions (Table 1). Importantly, during ER stress both ER-localized NOX4 and plasma membrane localized NOX2 can activate cell death in a ROS-dependent fashion (49, 50). A third crucial function of the ER, which may induce oxidative stress, is the electron transfer chain of the “microsomal monooxygenase” (MMO), a multi-enzymatic system localized in the ER of most animal tissues whose main function is to oxygenate hydrophobic exogenous (e.g., xenobiotics) and endogenous substrates (e.g., arachidonic acid, steroids) (51). Intriguingly, a large part of the reducing substrates are employed by eukaryotic P450 enzymes to generate ROS, while the efficiency and coupling of P450 enzymes are regulated by cofactors, such as cytochrome b_5_, or by post-translational modifications, such as phosphorylation (52). Finally, hormones (e.g., glucagon, glucocorticoids and thyroid hormones), cytokines (e.g., interferon and tumor necrosis factor), and apoptotic stimuli may modulate MMO-dependent ROS production (52, 53).

**Table 1:** **NADPH oxidase family members**.

Family member	Regulator	Biological function	Reference
NOX1	NOXO1; NOXA1; RAC1; p22^phox^	Host defense; growth factor and hormone (e.g., AngII) signaling.	(54–56)
NOX2	P47^phox^; p67^phox^; p40^phox^; Rac1; p22^phox^	Oxidative burst; ER-stress-induced apoptosis; AngII signaling.	(50, 57, 58)
NOX3	NOXO1; p22^phox^	Development of otoconia crystals.	(59)
NOX4	p22^phox^	ER-stress-induced apoptosis.	(49)
NOX5	Ca^2+^; phosphorylation	Spermatozoa motility; endothelial cell proliferation and capillary formation; smooth muscle cell proliferation and migration.	(60–62)
DUOX1	Ca^2+^	Thyroid hormonogenesis.	(63)
DUOX2	Ca^2+^	Host defense; iodination of thyroid hormone.	(56, 64)

**Figure 2:**
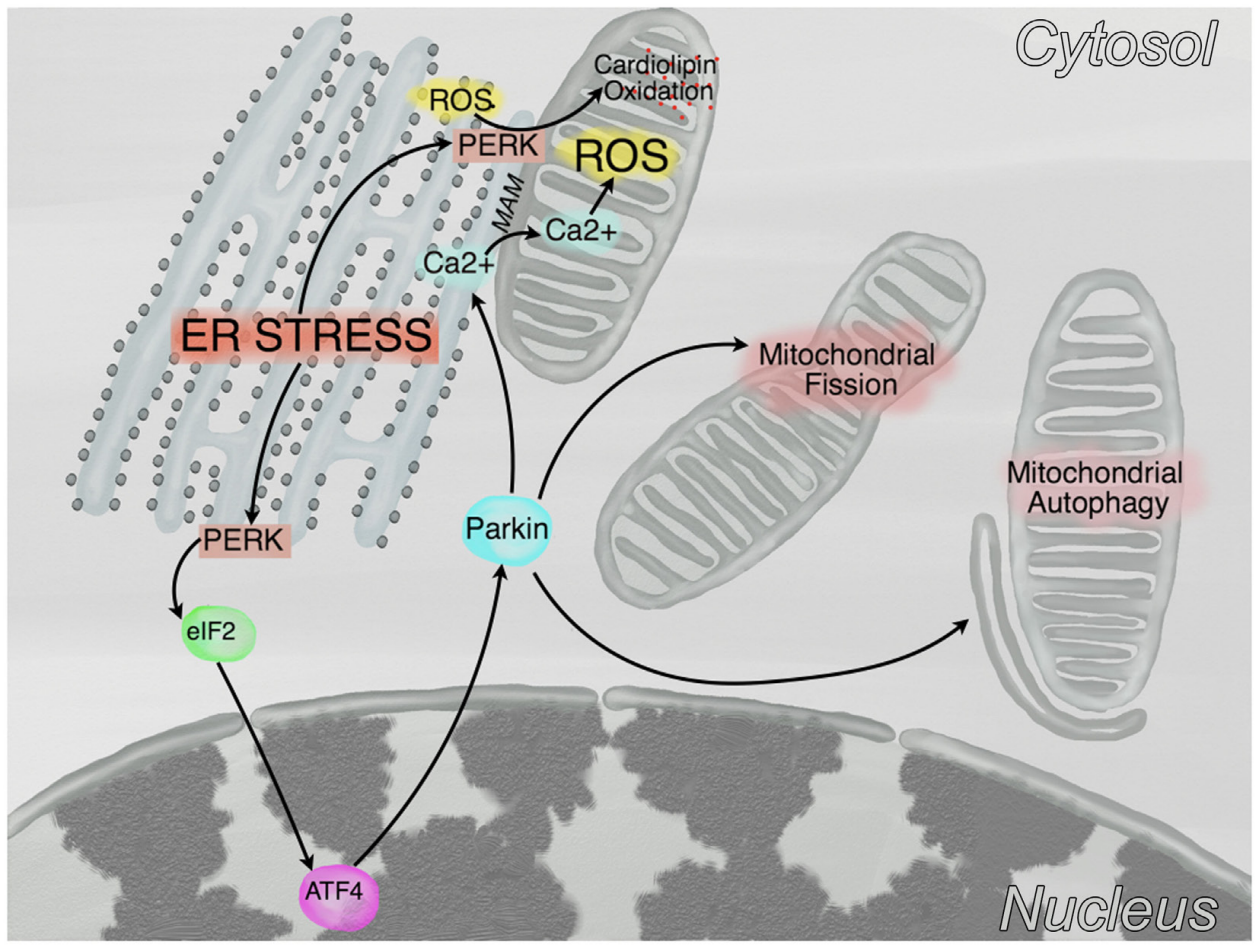
**Crosstalk between endoplasmic reticulum (ER) and mitochondria**. In conditions of ER stress, PKR-like ER kinase (PERK) phosphorylates eIF2α which, in turn, reduces protein translation and promotes the expression of the transcription factor ATF4. This latter, subsequently promotes the transcription of Parkin, a protein that stimulates mitochondrial fission, mitochondrial autophagy, and the transfer of calcium from the ER to the mitochondria at the mitochondria-associated membranes (MAM). Furthermore, a kinase independent function of PERK promotes the tethering of the ER to mitochondria at the MAM and the oxidation of cardiolipin.

Although *mitochondria* have been identified since many years as one of the major sources of ROS, it has been recognized only more recently that, as shown in Figure 2, in case of ER stress, a cross talk between ER and mitochondria promotes mitochondrial dysfunction [see (65) for a detailed review]. In this regard, ATF4, a transcription factor involved in the UPR response, increases the expression of Parkin, a ubiquitin ligase that regulates mitochondrial fission [see discussion below (66)], bioenergetics—by favoring transients of Ca^2+^ transfer from ER to mitochondria (67)-, and mitophagy (68), while PERK (double stranded RNA-dependent protein kinase (PKR)-like ER kinase), another mediator of the UPR, is enriched at the mitochondria-associated ER membranes and favors the propagation of ROS from ER to the mitochondria (69). A major role in this cross-talk is played by Ca^2+^, which is mainly stored in the ER, where it is imported by the sarcoplasmic/endoplasmic reticulum ATPase and is released via both the inositol 1,4,5-triphosphate receptor (IP_3_R) and the ryanodine receptor (RyR, in excitable cells as neurons and myocytes). Ca^2+^ is released from the ER during stress, enters mitochondria and increases both oxidative phosphorylation (70) and (possibly) ROS generation [critically reviewed in Ref. (71)]. Conversely, ROS themselves can alter calcium homeostasis by regulating IP_3_R (72) and RyR (73) function. Finally, recent evidences indicate that mitochondria do not exist permanently as single entities, but they rather form dynamic networks, exchanging proteins, lipids and mtDNA by fusion and fission cycles. Although mitochondrial fusion and fission regulate mitochondrial dynamics, it is unclear why do these processes happen. It has been postulated that mitochondrial fusion could dilute damaged mtDNA with wild type mtDNA, while fission could separate dysfunctional mitochondria to be sent to the autophagy/lysosomal pathway (74).

*Cell senescence* may affect the above-cited pathways at multiple levels. In this regard, p53 and mechanistic target of rapamycin (mTOR) signaling play a central role. mTOR, whose over-activation has been considered to act as a molecular switch between cell quiescence and senescence (75), overloads the ER, possibly activating the UPR. Although this latter activates autophagy and stimulates mitophagy (65), mTOR exerts opposing effects, inhibiting autophagy and lysosomal biogenesis (76), possibly leading to the accumulation of dysfunctional mitochondria. In line, with a systems biology approach, Dalle Pezze et al. recently demonstrated that the dual inhibition of ROS and mTOR signaling was required to partially reduce senescence-induced mitochondrial dysfunction and DNA damage in early stages of irradiation-induced cell senescence (27). Consistently, oncogenic RAS and oxidative stress activate NOX1 and NOX4, promoting autophagy and senescence, via p38 MAPK, p53, and p16^INK4A^ pathway in a ROS-dependent fashion (77, 78). Finally, cell senescence depresses mitochondrial fusion and fission events, thus leading to the accumulation of dysfunctional mitochondria (27).

With specific reference to the cardiovascular system, mTOR activation may occur as a consequence of both increased glucose 6 phosphate availability in overloaded hearts (79), and of the accumulation of branch chain amino acids, a condition that occurs with heart failure (80).

### Mechanisms of Redox Signaling

With specific regard to the mechanisms of redox signaling, several models have been hypothesized, according to which ROS signal either directly by oxidizing (usually) crucial *cysteine residues of signaling proteins* or indirectly via either hydroperoxidation of peroxiredoxins (PRDX) or modulation of the thioredoxin (TRX) redox status [reviewed in Ref. (81)]. Protein tyrosine phosphatases (PTPs) are the best characterized and most studied redox regulated signaling proteins. PTP protein family includes classical tyrosine phosphatases and dual-specificity phosphatases (82). These latter comprise members that can dephosphorylate either phosphoserine and phosphothreonine residues or phosphatidylinositol (3,4,5)-triphosphate [catalyzed, this latter event, by phosphatase and tensin homolog (PTEN)] (83). When critical cysteine residues in the active site are oxidized, the phosphatase activity is inhibited, promoting kinase signaling. Sensitivity to specific oxidants (e.g., H_2_O_2_ vs. ${\text{O}}_{2}^{\cdot -}$) and restriction of oxidative events to cellular microdomains provides target specificity [see Ref. (84) for review]. More recently, it has been shown that the activity of protein kinases too may be modulated (both positively and negatively) in a redox-dependent fashion. Specifically, this process was first described in Src and, subsequently, in many kinases that are involved in cardiovascular physiology and function, including Src family kinases, receptor tyrosine kinases (e.g., insulin receptor, epidermal growth factor and platelet derived growth factor receptors), c-Abl, Akt2, cAMP- and cGMP-dependent protein kinases (i.e., PKA and PKG, respectively), ERK2, Jnk2, p38 MAPK, Ca^2+^/calmodulin-dependent protein kinase II (CaMKII), ataxia-teleangectasia mutated protein kinase (ATM), and ASK1 (85).

To function in a reversible fashion, redox signaling requires, in analogy with phosphatases in protein kinase signaling, the existence of systems able to reduce oxidized targets. For example, critical cysteine residues can be oxidized to sulfenic, sulfinic, or sulfonic acid derivatives, whose reaction with neighboring amino acid residues can form intramolecular, reversible bonds, that can promote conformational changes of active sites and inhibition of the protein activity (84). To prevent or reverse these processes, the cell is equipped with *antioxidant molecules* (e.g., glutathione) and *antioxidant enzymes*, which show substrate specificity (e.g., superoxide dismutases reduce only superoxide, while catalase and PRDX only act on H_2_O_2_). Intriguingly, although initially considered only as ROS scavengers, these systems are now considered to act as sensors, involved in redox signal transduction. Among the most prominent antioxidant systems, we must acknowledge the TRX system, that catalyzes electron transfer from NADPH to TRX via thioredoxin reductase [extensively reviewed in Ref. (86)]. Proteins pertaining to the TRX family (e.g., TRX1, TRX2, glutaredoxins, PDIs, and quiescin-sulfhydryl oxidase), in turn, reduce substrate proteins—including other reductive enzymes (86). In addition, TRX proteins have been involved in the modulation of protein function by the covalent binding of NO to target cysteines (i.e., S-nitrosylation). More than a thousand proteins have been shown to be S-nitrosylated *in vivo*, many of which are involved in the pathogenesis of degenerative diseases (e.g., Parkin and Ryanodine Receptors) (87). Intriguingly, TRX1 can be nitrosylated on Cys^73^ by S-nitrosoglutathione, once a disulfide bond is formed between its Cys^32^ and Cys^35^ and the TRX1 disulfide reductase and denitrosylase activities are inhibited. In this condition, TRX1 can inhibit apoptosis promoting the *trans-*S-nitrosylation of Caspase3 (88). This finding indicates that TRX1 can either promote *trans-*S-nitrosylation or de-nitrosylation, depending on the redox status of the cell. Consequently, the regulation of TRX reductase activity is crucial for modulating its different functions. Among the known regulators of TRX reductase, we can include the transcription factor nuclear factor (erythroid-derived 2)-like 2 (Nrf2), selenium, and, most importantly for our discussion, caveolin 1, a structural component of *caveolae*, whose expression is induced by p38 MAPK (89, 90). Intriguingly, we recently discussed the involvement of caveolin 1 in cardiac stem cell senescence (91). Among the reductive enzymes regulated by TRX, the PRDX family of reductive enzymes is responsible for scavenging H_2_O_2_ and are involved, together with TRX and interacting proteins, in regulating apoptosis via ASK1 and proliferation via PTEN (86). Furthermore, oxidation-reduction cycles of PRDX are nowadays considered to be a universal marker of circadian rhythm and provide a clue on the connection between the cellular redox state and the circadian clocks (92). Intriguingly, it has been shown that cell senescence impairs circadian rhythmicity and, conversely, that an impaired expression of clock genes is associated with aging, inducing cell senescence in a redox-dependent fashion (92, 93).

A special mention in our discussion should be given to the major role played by ROS in the *regulation of autophagy*. In line, redox signaling modulates the autophagic pathway at several levels. It may be briefly summarized that ROS induce autophagy, which, in turn, reduces the production of ROS. Both peroxisome derived H_2_O_2_ and mitochondria derived ${\text{O}}_{2}^{\cdot -}$ are involved in this process (94, 95). Importantly, a blockade of mitochondrial autophagy promotes the accumulation of damaged, ROS generating mitochondria. This event in turn triggers the activation of the nucleotide-binding oligomerization domain, leucine-rich repeat and pyrin domain containing 3 (NLRP3) inflammasome, thus promoting the release of cytokines that spread cell senescence and inflammation in a paracrine fashion (96).

Although *lipids* can be oxidized by lipoxygenases, cyclooxygenases, and cytochrome P450 to generate signaling molecules, ROS (e.g., hydroxyl radical—HO^•−^ and hydroperoxyl—${\text{HO}}_{2}^{.-}$) produced in less controlled conditions may also cause peroxidation of lipids containing carbon-carbon double bonds, such as polyunsaturated fatty acids (PUFA), generating lipid peroxyl radicals and hydroperoxides (97). Malondialdehyde (MDA) and 4-hydroxynonenal (4-HNE) are among the best characterized secondary products of lipid peroxidation. MDA can be produced either by enzymatic processes during the metabolism of PUFA, such as the biosynthesis of thromboxane A_2_, or by non-enzymatic reactions (98). Intriguingly, recent data indicate that MDA, not only can promote intramolecular or intermolecular crosslinking, DNA damage or mutation (99), but can also act as a signaling molecule that regulates, for example, the glucose-stimulated insulin secretion in murine islets (100) or inhibit cardiac contractility via p38 MAPK (101). 4-HNE as well may be produced during enzymatic metabolism (i.e., as a consequence of the action of arachidonate 15-lipoxygenase) or by non-enzymatic oxidation of ω-6 PUFA (102). The effects of this hydoxyalkenal have been extensively studied and reviewed (98). Specifically, 4-HNE has been shown to regulate stress responsive transcription factors (among which NRF2, NF-κB, and peroxisome-proliferator-activated receptors) that modulate the expression of antioxidant proteins, inflammation, and metabolism; moreover, it may modulate the function of MAPK (e.g., p38 and JNK) and of protein-kinase C. Of particular interest for our discussion is the role played by 4-HNE in cell senescence, since it may inhibit the expression of the catalytic subunit of telomerase (hTERT) (103) and activate p53 (104).

Finally, ROS can oxidize *nucleic acids*. Whereas most of the attention has been dedicated to the study of nuclear and mtDNA oxidation, recent data indicate that, in many pathological conditions (e.g., neurodegenerative diseases, atherosclerosis, and diabetes), RNA can be oxidized too (105–108). RNA oxidation stalls the translational machinery, thus reducing the rate of protein synthesis (109). Intriguingly, several reports indicate that RNA oxidation is not a random process, but affects genes involved in specific functions, such as: protein and ATP biosynthesis, electron transport chain, tricarboxylic acid cycle, glycolysis, protein folding and UPR, enzymatic anti-oxidation, and autophagy/lysosome pathway-mediated degradation (106, 107).

## Redox Signaling and APE1/Ref

**Figure 3:**
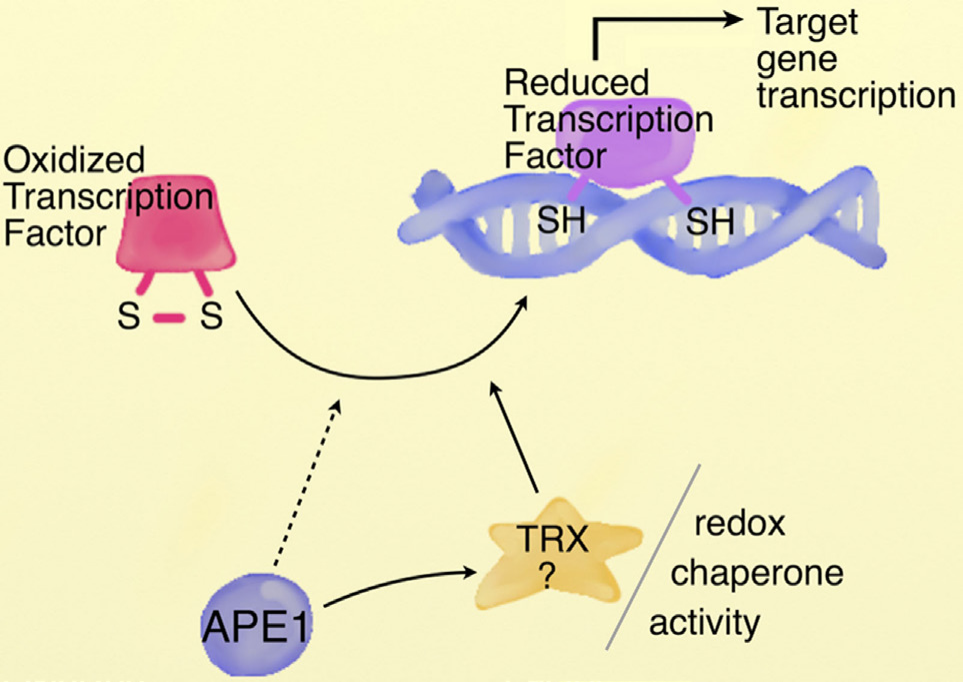
**APE1 redox chaperone activity**. Schematic representation of APE1 redox chaperone activity. Cysteine residues located within the DNA binding domain or regulatory domain of transcription factors are maintained in a reduced state by APE1, involved in a redox cycle with reducing molecules as TRX. The detailed redox mechanism by which APE1 controls the activity of transcription factors needs further investigation. [Image modified, with permission from the copyright holder, from Tell et al. (114) *Cell Mol Life Sci*].

As previously stated, beside their toxic effects, ROS play a critical role as signaling molecules, altering the cellular and extracellular redox state, in a broad variety of cellular processes as cellular proliferation and survival, DNA damage response and gene regulation. A large body of research demonstrates a general effect of oxidative stress on gene expression via direct modulation of transcription factors activity, regulation mediated by the redox state of thiol groups exposed by critic cysteine residues, located within the DNA binding domain of the transcription factors. At first, independent studies have identified APE1, the main AP-endonuclease of BER pathway of DNA lesions, as a factor able to stimulate the binding activity of several transcription factors through a redox-dependent mechanism (110). Since this first report, several transcription factors, both ubiquitous [e.g., NF-κB, Egr-1, p53, CREB, hypoxia inducible factor 1α (HIF-1α)] and tissue specific (e.g., TTF-1 and Pax proteins), with opposite effects on cell fate, have been reported to be modulated by APE1 (111). Interestingly, it is likely that APE1 could select, in response to specific stimuli, which transcription factors should be regulated, binding to them with different efficiencies. It has been proposed that APE1 might regulate the binding activity of transcription factors via regulation of the cysteine state, located in the DNA binding domain or domains critical for their activity, maintaining them in a reduced state by direct interaction (Figure 3) (112–114). Three possible mechanisms, not mutually exclusive, might explain the APE1 redox chaperone activity. In the so-called recruitment model, APE1 may facilitate the reduction of the transcription factors by bridging them to the reducing molecule (GSH or Trx). According to the conformational change model, instead, APE1 may induce a conformational change in the target factor, rendering accessible the cysteine residues otherwise buried in the structure. Finally, according to the oxidation barrier model, APE1 may stabilize the reduced state, preventing the oxidation, via formation of hydrogen bond with thiol groups (113). The detailed molecular steps for the activation of the transcription factors by APE remain to be largely investigated. Up to now, however, none of the APE1 cysteine residues has been found in a C-X-X-C motif, common to most redox regulatory factors, such as TRX, which is implicated in the redox APE1 cascade by restoring APE1 in a reduced form (115). Several lines of evidence propose Cys65 as the redox-active site for APE1 (116, 117); however, the three-dimensional structure model demonstrates that none of the APE1 cysteine residues is localized in the right position and distance for forming a disulfide bond with Cys65, a prerequisite for resolving activity of redox factors. Moreover, both Cys65 and Cys93, residues identified as responsible for the redox regulation, are buried within the APE1 structure, being surface inaccessible, whose activities may be influenced by partial APE1 unfolding (118). Recently, Georgiadis and coworkers have proposed a new model based on current knowledge, suggesting that APE1 is a unique redox factor with properties distinct from those of other redox factors. In this model, (i) Cys65 acts as nucleophile for the reduction of the disulfide bond in the target factor, (ii) Cys93 operates as resolving residue, and (iii) APE1 undergoes a significant partial unfolding to expose a third cysteine residue, probably Cys99, to facilitate the redox reaction (118). Further works are needed to delineate the precise molecular mechanism of the reaction.

The localization of APE1 is predominantly nuclear, although the protein is also present within the mitochondrial compartment as essential component of the mtBER pathway (119). Notably, the mechanisms regulating APE1 intracellular trafficking are still largely unknown. In 2011, Li et al. demonstrated that the APE1 mitochondrial localization signal resides within residues 289–318 of the C-terminus, which are normally masked by the N-terminal domain. This suggests the necessity of specific and regulated mechanisms of protein unfolding–refolding to ensure proper APE1 localization. Additional studies identified residues Lys299 and Arg301 as critical residues for APE1 interaction with Tom20 and consequently for the mitochondrial translocation of the protein (120). Further advancement in the characterization of the mitochondrial residence of APE1 was brought about by our recent work, where we discovered a role for the redox-based MIA pathway to promote the mitochondrial translocation of APE1 (121). The Mitochondrial intermembrane space import and assembly protein 40 (Mia40) and augmenter of liver regeneration represent the central components of the MIA pathway; an import machinery that uses a redox system to drive cysteine-rich proteins into the mitochondrial compartment (122, 123). Recently, we demonstrated that human Mia40 is able to interact with and bind APE1 by forming a disulfide bridge between the residues Cys93 on APE1 and Cys55 on Mia40. Importantly, Cys53 and Cys55 constitute the catalytic cysteine-proline-cysteine (CPC) motif of Mia40. Mutation of APE1 at Cys93 negatively affects the capacity of the protein to accumulate in an active form within the mitochondria leading to increased levels of mtDNA damage (121). The inner membrane complex responsible for the translocation of APE1 into the matrix compartment has still to be identified, as well as the role of APE1 within mitochondria has to be clarified. Back in 1995, Barzilay et al. hypothesized for the first time the existence of non-canonical functions for APE1, suggesting a potential involvement of APE1 in the RNA metabolism and measuring its RNaseH-like activity (124). Only later, evidence of APE1 association with ribosomes in the cytoplasm of different cell types suggested that APE1 could also bind to RNA molecules *in vivo*. In addition to the direct interaction with RNA molecules, APE1 was also found to interact with other factors involved in RNA metabolism such as YB-1 and hnRNP-L. Altogether, these observations suggested a profound involvement of APE1 in the RNA metabolism, but until recently it was not clear what kind of activity APE1 could exert on RNA molecules (125). We first demonstrated that APE1 has endonuclease activity not only on AP-site containing double-stranded DNA but also on single-stranded DNA and RNA (125), suggesting a potential cleansing role for APE1 on abasic RNA. Unlike DNA molecules, RNA is mostly single stranded, with bases not protected by base pairing or by specific proteins. These features can result in increased susceptibility to oxidative insults. If let unrepaired, damage to RNA can lead to altered pairing, resulting in ribosomal dysfunction and erroneous translation thus significantly affecting the entire protein synthesis mechanism. Interestingly, oxidative damage to RNA molecules, with either coding (mRNA) or non-coding (rRNA and tRNA) functions, has recently been associated with the occurrence of several human degenerative diseases (125). Findings pointing to a previously overlooked “quality control” function for APE1 on oxidized RNA are therefore surprising and open interesting perspectives to our comprehension of cellular biology.

### Redox Signaling and APE1/Ref in Stem Cell Biology

More than a decade of research has indicated the relevance of oxidative stress and, more recently, of redox signaling in stem cell biology. In this regard, of particular relevance is the demonstration of both: hypoxia as a common theme of stem cell niches (126, 127), and the modulation of stem cell self-renewal, differentiation and apoptosis via a “ROS rheostat” (128). These two factors are intertwined, since the prevailing hypothesis is that hypoxia stabilizes HIF 1α, a transcription factor that coordinates the adaptation to low oxygen tension, favoring glycolytic over an oxidative metabolism and promoting a quiescent, primitive phenotype (129). Conversely, mitochondrial activity and ROS levels increase as stem cells differentiate (129, 130). These findings support a role for redox signaling in regulating stem cell differentiation processes.

In this regard, it is emerging that *ATM* kinase regulates stem cell quiescence, protecting them from stress (131). This protein, that is not only activated by double strand DNA breaks, but also by hypoxia and oxidative stress, reduces ROS production, possibly by inhibiting mTOR (thus stimulating autophagy) via either the AMP kinase/tuberous sclerosis complex 2 axis or the HIF1 α one (132–134). Moreover, an ATM-mediated phosphorylation of BH3-interacting domain death agonist (BID) can regulate mitochondrial ROS production and promote hematopoietic stem cell activation (131).

A similar role in stem cell integrity is played by *p53*. Consistently with its role in stem cell homeostasis, p53 can be stabilized by hypoxia, and its transcriptional activity can be modulated in a redox-dependent fashion (135, 136). Specifically, under steady state (or low stress) conditions, p53 promotes stem cell quiescence over self-renewal and provides protection against oxidative stress (e.g., inducing the expression of glutathione peroxidase or SOD2), while following acute genotoxic stress, it may be activated (e.g., via ATM), promoting cell cycle arrest, DNA repair, or apoptosis (136, 137).

*APE1* deserves a special mention in our discussion. Indeed, it has been demonstrated that APE1 is more involved in regulating p53 basal levels, than in modulating the activation of p53 in response to DNA damage (138). Consistently, it has been shown that: APE/Ref-1 is expressed in undifferentiated embryonic and adult stem cells (i.e., primitive cells isolated from bone marrow, heart, adipose tissue, and dental papilla), it protects them from oxidative stress, and, in some cases, it was shown to prevent their differentiation (130, 139–141). Conversely, it has been shown that senescence of mesenchymal stem cell is associated with a decreased expression of APE1 (even in the presence of high intracellular ROS levels), while the forced expression of APE1 was sufficient to rescue the senescent phenotype (142). In line, the forced expression of APE1 in Sca-1-positive cardiac progenitors is able to increase their apoptosis resistance and to improve their potential to promote myocardial infarction repair (143). However, APE1 is also involved in the differentiation process of stem cells toward the cardiac, neural and hemangioblast phenotype, being upregulated by the increased intracellular ROS levels that characterize the differentiation processes. Intriguingly, the redox function of APE1 can enhance, repress or direct stem cell differentiation toward specific cell types (e.g., neurons and cardiomyocytes) (130, 139, 140).

## Redox Signaling in Cardiovascular Disease and Heart Failure

As anticipated, redox signaling plays a relevant role in the cardiovascular system too. In line, hypoxic cardiac stem cell niches have been identified *in vivo* by independent groups (144, 145). Kocabas et al. showed that the epicardium and subepicardium are the cardiac regions with the lowest vascular density, where HIF1α expressing cells are localized. This latter transcription factor would promote a glycolytic metabolism and modulate the differentiation of primitive cardiac cells (126). With an alternative approach that employed the hypoxic probe pimonidazole, Sanada et al. showed the coexistence of normoxic and hypoxic niches containing c-Kit^+^ cells in adult mouse hearts. Intriguingly, they also demonstrated that aging is associated with an increased frequency of hypoxic niches containing quiescent and senescent stem cells (145). However, according to recent reports, the *in vitro* expansion of c-Kit^+^ cells in an hypoxic environment (0.5% O_2_) keeps cardiac stem cells more quiescent, reducing their rate of cell senescence, with respect to standard cultures kept at room air (21% O_2_) (146).

Concerning cell differentiation, it has been shown that different stimuli (e.g., electric field stimulation, PI3-kinase signaling) can increase cardiomyocyte differentiation of pluripotent cells by increasing intracellular ROS (147, 148). In this process, NOX4 is considered to be crucial, promoting the nuclear translocation of MEF2C in a p38MAPK-dependent fashion (144). However, the level of ROS must be strictly controlled, since, when excessively high, they may inhibit cardiomyocyte differentiation, too (144, 149). Consistently, it has been also shown that the modulation of intracellular ROS, paralleling mitochondrial maturation and closure of the mitochondrial permeability transition pore, promotes the differentiation of embryonic cardiomyocytes (150). Intriguingly, while undifferentiated pluripotent cells are characterized by a highly unsaturated metabolome, oxidized metabolites (e.g., acyl-carnitines) promote cardiomyocyte differentiation (151).

Regarding redox signaling in differentiated cells, a peculiar place is occupied by endothelial cells (ECs), whose fate is regulated by redox sensitive molecules (that include the transcription factors forkhead box O -FoxO-, Nrf2 and HIF, as well as AMPK, PTEN, VEGFR, and eNOS) [reviewed in Ref. (152)]. Being at the interface with blood, EC are exposed to oxygen, metabolites, and mechanical deformation (i.e., shear stress) that may perturb their redox balance. Furthermore, EC play crucial roles in cardiovascular homeostasis through the release of NO^•^ (which is a labile free radical itself). Therefore, although redox signaling is vital for EC, they are in contact with exogenous sources of oxidative stress toward which they must be protected by means of an antioxidant reserve. In line, cardiovascular risk factors (e.g., hyperglycemia, smoking) are characterized by their ability to increase ROS generation and to decrease endothelial NO^•^ production (153). Going back to redox signaling in vascular cells, it has been shown that NOX1, 2, 4, and 5 are expressed in EC (60), where they exert important physiological roles. However, in pathologic conditions, the induction of endothelial NOX increases intracellular ROS levels, triggering the further production of free radicals from other sources, such as xanthine oxidase and endothelial nitric oxide synthase (154). Hyperglycemia, for example, can increase the expression of NOX1 in human aortic ECs, while inhibition of NOX1 in diabetic apolipoprotein-E (ApoE) deficient mice reduces atherosclerosis development (155). Hyperlipidemia too may stimulate ROS production by NOX. Accordingly, remnant lipoprotein particles or oxidized LDL upregulate NOX2 expression and increment ROS production (156, 157), while NOX2^−/−^ ApoE^−/−^ double knockout mice are protected against endothelial dysfunction and development of atherosclerotic lesions in descending aorta (158). Concerning hypertension, vascular NOX are activated by different pro-hypertensive factors. Among these, angiotensin II (AngII) increases NOX2 expression and function, while deletion of NOX1 and NOX2 in hypertensive mouse models is protective (159, 160). Intriguingly, although patients carrying hereditary deficiency of NOX2 are affected by chronic granulomatous disease, they also show increased flow-mediated arterial dilation and increased NO bioavailability (161). Conversely, NOX4 has been shown to exert protective effects on atherosclerosis development, in mouse models (162). The discordant behavior of NOX4 with respect to NOX1 and 2 has been attributed to the unique features of this enzyme, since it is constitutively active and produces mainly H_2_O_2_ (162). Moreover, NOX4 is induced by hypoxia and can stimulate angiogenesis activating eNOS(163). However, experimental evidence indicates that NOX4 may be involved in plaque instability too. Specifically, authors observed that, while during the early phases of plaque formation NOX1 was induced, during the late phases, NOX4 prevailed (164). Concerning vascular smooth muscle cells (SMCs), they express NOX4, together with NOX1 and 5 (153). The latter has been identified only recently and its characterization has been hampered by the absence of an ortholog in mice (62). NOX5 is activated by thrombin, PDGF, AngII, and endothelin 1 and regulates SMC proliferation and migration, processes that are critically involved in atherosclerosis (62). Importantly, NOX5 levels are increased, both in EC and SMC, in coronary artery disease (165). However, at present its pathophysiological role is still unclear (166). Concerning the involvement of senescence processes in the development of atherosclerosis, it has been recently demonstrated that senescent foam cells, ECs, and SMCs can be identified in advanced atherosclerotic plaques. Furthermore, the selective removal of senescent cells, by means of an inducible suicide gene, significantly reduces the formation of atherosclerotic lesions in atherosclerosis-prone Ldlr^−/−^ mice (167). Important stressors with a relevant role in atherosclerosis (e.g., AngII, advanced glycation end products -AGE-, inflammatory molecules, and NOX4 activation) are known inducers of vascular cell senescence (164, 168). Moreover, cell senescence activates a vicious cycle characterized by a nucleus to mitochondria cross talk that can eventually perturb mitochondrial dynamics and reinforce cellular senescence (169). Furthermore, dysfunctional mitochondria may activate the NLRP3 inflammasome, spreading vascular cell senescence paracrinally (170).

Redox signaling is a known mediator of cardiac adaptation to different physiological and pathological stimuli, that act both in the acute adaptation to damage and in cardiac remodeling (171). The major oxidant sources in myocytes are, similarly to vascular cells, NOX, xanthine oxidase, uncoupled NOS, and mitochondria (including MAO) (171–173). Both NOX2 and 4 can be found in cardiomyocytes. However, the first one is localized at the plasma membrane and in T tubules and requires mechanical or agonist stimulation (i.e., G-protein coupled receptor agonists, cytokines and growth factors) to be activated (174). Conversely, NOX4 is localized in intracellular membranes (i.e., the ER and mitochondria), is constitutively active, and is regulated mainly at a transcriptional level (174). NOX2 generated ROS are causally involved in the development of AngII-induced hypertrophy(175), LPS-induced myocardial depression (176), adverse remodeling after myocardial infarction (177), and in alcohol-related cardiomyopathy (178). Contrariwise, NOX4 expression seems to be protective in cardiac overload, by increasing capillary density (in a HIF1α-dependent fashion), thus protecting against contractile dysfunction and cardiac dilation (179). The positive effects of NOX4 expression include the induction of Nrf2 expression (180), the promotion of autophagy via the PERK/eIF2α/ATF4 pathway (181), and the enhancement of fatty acid oxidation (182). However, according to other reports, chronic and high activation of NOX4 exerts negative effects following pressure overload (183) or phenylephrine stimulation, by promoting the exclusion of histone deacetylase 4 (HDAC4) from the nucleus, thus stimulating cardiac hypertrophy (183). Antioxidant systems too are involved in hypertrophy, as shown in mice overexpressing either TRX1 or its dominant negative form (184). The mechanism of TRX1-mediated cardioprotection involves the transnytrosilation of ≈40 target proteins, including chaperones, metabolic-, structural-, redox-, and ribosomal-proteins and translation regulators (88). Furthermore, TRX1 can protect against hypertrophy in a redox-dependent fashion by regulating the nucleocytoplasmic shuttling of HDAC4 (185). In line, several transcription factors that are involved in cardiac remodeling (including GATA4, MEF2, NFAT, SRF, and NFκB) are under the control of redox sensitive mechanisms. For example, MEF2 and NFAT are inhibited by HDAC4 (185, 186), GATA4 is activated by ROS via RhoA/ROCK and MEK/ERK (187), SRF is activated via MICAL-2/MRTF-A in a redox-dependent fashion, and NFκB can be activated via APE/Ref (114). Intriguingly, pathways linked to physiological hypertrophy too, such as the PI3K/Akt one, can be regulated in a redox-dependent fashion (in this specific case by PTEN) (188). Finally, myocyte contractility, calcium dynamics, and excitation contraction coupling too can be modulated by redox sensitive molecules, such as PKA, CaMKII, RyR2, phospholamban, and ion channels (e.g., L-type calcium channels) (174).

A special mention deserves the involvement of APE1 in cardiovascular pathophysiology. Specifically, APE1 reduces apoptosis in ECs exposed to hypoxia and TNFα via NFκB-dependent and -independent mechanisms (189). Moreover, APE1 hemizygous mice are characterized by impaired endothelium-dependent vasorelaxation, reduced NO levels and hypertension. In fact, experimental evidence indicates that APE1 modulates Akt signaling and eNOS function via H-RAS (190). Moreover, E3330, a pharmacologic inhibitor of the redox function of APE1, impairs both the growth of tumor associated ECs and the differentiation of bone marrow derived cells into CD31^+^ expressing cells. These effects were mediated by the modulation of both H-RAS levels and of the DNA binding activity of HIF1α (191). Concerning atherosclerosis, it was shown that oxidative DNA damage markers and APE1 levels are increased in human atherosclerotic plaques (192). Elevated level of APE1 was also found in the serum of patients affected by coronary artery disease and were further incremented in the presence of myocardial infarction (193). This finding may be considered a compensatory mechanism, since it was also shown that APE1 gene transfer exerts a protective effect on atherosclerosis development, reducing SMC migration and neointima formation (194). A further protective effect of APE1 has been shown in ischemic preconditioning, where the nuclear accumulation of APE1 mediates cardioprotection (195). Similarly, APE1 was able to protect cardiomyocyte from death induced by miconazole, a cardiotoxic drug able to increase intracellular ROS levels (196). Intriguingly, the expression of *exo-3* (the *Caenorhabditis elegans* ortholog of APE1) declines with aging, while reduced levels of *exo-3* shorten the nematode lifespan (197). Although a decline in the induction of APE1 in response to oxidative stress was observed in the brain of aged rats (198), no data have been collected on the role of APE1 cardiovascular aging.

Finally, it is well recognized that the cardiac metabolism is altered during the development of cardiac pathology. Specifically, a reduction of the myocardial energy reserve (i.e., phosphocreatine/ATP ratio), an increase of the glucose uptake (and a decrease of the fatty acid uptake in overt heart failure), and, most importantly, an activation of the pentose phosphate pathway (PPP) characterize cardiac pathology and heart failure (199). Although the PPP is a source of NADPH and may be considered as a compensatory mechanism that is in place in heart failure, recent data suggest that an excessively active PPP may feed NOX enzymes and uncoupled nitric oxide synthase, paradoxically increasing oxidative stress (200). Conversely, Katare et al. have recently demonstrated that diabetes impairs the PPP and accelerates the dysfunction, senescence, and death of cardiac resident stem cells. Importantly, these alterations may be reversed by boosting the PPP (17, 201).

## Conclusion and Perspectives

Reactive oxygen species have a dual nature; they exert fundamental roles in signal transduction and in many biological processes, with a level of complexity that has been only partially delineated. However, they may exert harmful effects too, leading to cell death and senescence. In this regard, the emerging knowledge is that redox signaling is tightly regulated and compartmentalized, while oxidative stress emerges from the loss of this highly controlled architecture. Importantly, redox signaling is a crucial regulator of stem cell quiescence, self-renewal, and differentiation. Conversely, loss of controlled redox signaling (oxidative stress) can obliterate stem cell function and promote cell senescence of stem and differentiated cells, two conditions that have been associated with the progressive loss of tissue renewal and reparative reserve that characterize aging. Cardiac stem cells are not immune from these pathological processes, becoming dysfunctional and unable to effectively repair cardiac damage with organism aging and pathology. mTOR signaling, which associates with cardiac stem cell senescence, may affect redox signaling at multiple levels, overloading the ER, and inhibiting both lysosomal function and autophagy. Therefore, innovative interventions aimed at restoring proper redox signaling in primitive cells have the potential to reverse or attenuate age-associated stem cell dysfunction. In this regard, although the functional manipulation of the multifunctional protein APE/Ref-1 in cardiac stem cells has been only recently experimented, it is a promising intervention, that deserves a deeper investigation.

## Author Contributions

DC: wrote the manuscript, AA: revised the manuscript, SS: revised the manuscript, CC: revised the manuscript, CL: revised the manuscript, GT: wrote the manuscript, and AB: wrote the manuscript.

## Conflict of Interest Statement

The authors declare that the research was conducted in the absence of any commercial or financial relationships that could be construed as a potential conflict of interest. The reviewer, PD, and handling editor declared their shared affiliation, and the handling editor states that the process nevertheless met the standards of a fair and objective review.
